# Calbindin-Expressing CA1 Pyramidal Neurons Encode Spatial Information More Efficiently

**DOI:** 10.1523/ENEURO.0411-22.2023

**Published:** 2023-03-13

**Authors:** Liqin Gu, Minglong Ren, Longnian Lin, Jiamin Xu

**Affiliations:** 1 Institute of Brain Functional Genomics, East China Normal University, Shanghai 200062, China; 2 New York University - East China Normal University Institute of Brain and Cognitive Science at NYU Shanghai, Shanghai 200062, China; 3 Tongji University Brain and Spinal Cord Clinical Center, Shanghai 200062, China

**Keywords:** firing pattern, hippocampus, pyramidal neuron, ripple, spatial coding, theta

## Abstract

Hippocampal pyramidal neurons (PNs) are traditionally conceptualized as homogeneous population. For the past few years, cumulating evidence has revealed the structural and functional heterogeneity of hippocampal pyramidal neurons. But the *in vivo* neuronal firing pattern of molecularly identified pyramidal neuron subclasses is still absent. In this study, we investigated the firing patterns of hippocampal PNs based on different expression profile of Calbindin (CB) during a spatial shuttle task in free moving male mice. We found that CB+ place cells can represent spatial information more efficiently than CB− place cells, albeit lower firing rates during running epochs. Furthermore, a subset of CB+ PNs shifted their theta firing phase during rapid-eye movement (REM) sleep states compared with running states. Although CB− PNs are more actively engaged in ripple oscillations, CB+ PNs showed stronger ripple modulation during slow-wave sleep (SWS). Our results pointed out the heterogeneity in neuronal representation between hippocampal CB+ and CB− PNs. Particularly, CB+ PNs encode spatial information more efficiently, which might be contributed by stronger afferents from the lateral entorhinal cortex to CB+ PNs.

## Significance Statement

Pyramidal neurons (PNs) in the hippocampus show heterogeneity along the longitudinal axis. But deep and superficial layer PNs are difficult to identify with traditional tetrode recordings. Combining with optogenetic tools, we were able to identify and record ephys patterns of Calbindin (CB)+ PNs in free moving mice. We found that CB+ place cells represent spatial information more efficiently in a spatial shuttle task, with more spikes fired inside than outside of place fields, and carrying more spatial information per spike compared with CB− peers. We also found heterogeneity of neuronal firing dynamics of hippocampal PN subtypes with respect to theta and ripple oscillations. These results suggest that we take into consideration such heterogeneity of PNs in future investigations of hippocampal function.

## Introduction

In the rodent hippocampus, cell bodies of CA1 pyramidal neurons (PNs) are densely packed in stratum pyramidale. People have treated hippocampal pyramidal neurons as homogenous with similar morphology, neural network connection and biophysical properties (Andersen et al., 2006). But recent studies have pointed out their heterogeneity in several aspects, including developmental origin (Cembrowski et al., 2016), molecular expression profile (Baimbridge et al., 1991; Klausberger and Somogyi, 2008; Dong et al., 2009), dendritic morphology (Bannister and Larkman, 1995; Graves et al., 2012), connectivity (Kohara et al., 2014; Lee et al., 2014; Masurkar et al., 2017), and electrophysiological (ephys) kinetics (Baimbridge et al., 1991; Valero et al., 2015).

Based on the relative somatic location of PNs to the border of stratum radiatum (S.R.), hippocampal CA1 PNs can be subdivided into superficial and deep layer neurons, with superficial neurons closer to S.R. Most neurons in the superficial layer of the dorsal CA1 area express Calbindin (CB), a Calcium-binding protein, while deep layer PNs did not. Superficial PNs also have more complex apical dendritic arborizations compared with deep PNs (Bannister and Larkman, 1995; Graves et al., 2012; Li et al., 2017). These two PN subtypes receive different excitatory synaptic inputs from several afferents including lateral, medial entorhinal cortex (MEC), and also CA2 (Kohara et al., 2014; Li et al., 2017; Masurkar et al., 2017). LEC preferentially projects to superficial PNs, while CA2 preferentially connects to deep PNs (Kohara et al., 2014; Li et al., 2017). They also differ in IPSC responses, induced either by CA3 or CA2 stimulation (Kohara et al., 2014; Valero et al., 2015). Deep PNs displayed larger IPSC responses, probably because of more inhibitory innervation by Parvalbumin positive interneurons on deep PNs than their superficial peers (Lee et al., 2014).

In addition, growing evidence revealed heterogeneity in neural activity of hippocampal PNs during hippocampus dependent behavior tasks. Using silicon probes, hippocampal PNs can be divided into superficial and deep layer PNs based on their somatic location relative to the middle of stratum pyramidale, which is defined by the largest amplitude of ripple oscillation (Mizuseki et al., 2011). Deep and superficial PNs showed significant differences in firing rate, phase-locked firing with hippocampal theta and ripple oscillations (Mizuseki et al., 2011; Stark et al., 2014; Danielson et al., 2016). A subset of deep PNs shifted their firing phase during rapid-eye movement (REM) sleep, while superficial PNs are consistently phase-locked with theta oscillation either during running or REM sleep (Mizuseki et al., 2011). During hippocampal ripple oscillations, superficial PNs are more active than deep PNs at least in anesthetized head-fixed animals (Valero et al., 2015). Deep PNs are more actively engaged in spatial navigation compared with superficial PNs in a two-photon Ca^2+^ imaging experiment (Danielson et al., 2016). In our previous study, we have shown that the direct projection from LEC to Calbindin-expressing (CB+) hippocampal pyramidal neurons plays an essential role in olfactory associative learning (Li et al., 2017).

Despite the progress, it remains unclear whether and how the anatomic and biophysical variations of PNs lead to their distinct roles in hippocampal functions. Specifically, the functional involvement of CB+ and CB− hippocampal PNs during different behavior tasks/states is still elusive. To answer this, we combined optogenetic tagging with multichannel *in vivo* recording and investigated the firing profiles of hippocampal CB+ and CB− PNs in freely moving mice under different behavior states.

## Materials and Methods

### Animals

Two lines of transgenic mice were used in this study, the Calb2-IRES-Cre line (Jax No.010774) and Ai35 mice (Rosa-CAG-LSL-Arch-GFP-WPRE, Jax No.012735). They were generously shared by our collaborator, Prof. Xiaohui Zhang from Beijing Normal University. The two mouse lines were crossed to selectively express Arch in CB+ neurons (Chow et al., 2010). This approach had also been reported in our previous study to identify different types of pyramidal neurons in the CA1 area of the hippocampus (Li et al., 2017). Ten male transgenic mice were used for *in vivo* electrophysiological recordings in this study. All procedures were approved by the Animal Advisory Committee at East China Normal University (No. AR201404009) and were performed in accordance with the National Institutes of Health *Guidelines for the Care and Use of Laboratory Animals*. Six mice with considerable good yields of neurons were used in further data analysis.

### Behavior training

There were two experimental setups in this study: the home cage recording of spontaneous neuronal firing across different behavior states and a U-shaped track for the recording of place cells. The home cage was a 28 × 45 cm^2^, 20-cm-high plastic box where the animal was housed with free access to food and water. The recordings typically last for several hours to cover different spontaneous behaviors, including quiet waking, REM sleep and slow-wave sleep (SWS). After the recording, the mouse was removed from the recording setup and kept in the same home cage for later experiments.

The U-shaped track has three arms, the left, the central and the right arm. The total length of the three arms is 114 cm with a uniform width of 5.5 cm. There were different visual cues along the track and a water port at the end of the left and right arm. Before training, mice were kept under a water restriction protocol. The animals were trained to run back and forth on the track to get a 10-ms water reward at the water ports. The amount of water reward was controlled by a solenoid valve, which was run by a program written with Labview (Labview 8.6, National Instruments). Track training and place cell recording were conducted in a dim environment surrounded by black curtains.

### Drivable optrode

An optrode was made of a 200-μm diameter optic fiber [numerical aperture (NA) = 0.39; Thorlabs], and surrounded by eight tetrodes. The optic fiber was later connected to a laser stimulator for optogenetic stimulation. The tips of the tetrodes extended ∼0.5 mm beyond the tip of the optical fiber. The optrode bundle was attached to a set of screws and nuts which can be driven by rotation of the screw, with each full turn corresponding to 280 μm in depth penetration. The detail of the assembly of the microdrive can be found from our previous study (Lin et al., 2006). A 64-channel microdrive with two optrode bundles was used in one mouse, each optrode targeting one hemisphere of the hippocampus. The other five mice were implanted with a 32-channel optrode (eight tetrodes).

### Surgery

The detailed procedure of the optrode implantation surgery can be found from our recently publication (Ma et al., 2020). In short, the scalp of the mouse was removed after anesthetization (pentobarbital sodium, 40 mg/kg bodyweight) and then mounted onto the stereotactic frame (Stoelting). Body temperature of the mouse was closely monitored and kept constant by a thermoregulation device (FHC). Multiple screws were mounted onto the skull (avoiding the hippocampal area), serving as the foundation for dental cement. One of the screws that were mounted above the cerebellum also served as ground. One or two craniotomies were made above the left and right hippocampus at these positions (in mm): −2.3 AP, +2.0 ML. After removal of the dura, the microdrive was slowly lowered into the brain so that the optrodes reached a depth of ∼0.9 mm from the cortical surface. After the optrode insertion, the microdrive was secured on the skull with dental cement. We wrap a piece of copper mesh around the entire microdrive, serving as a Faraday cage and also protecting the microdrive from potential scratching damage. After surgery, the mouse was housed in the home cage with free access to food and water, on a 12/12 h light/dark cycle. The animals were allowed to recover for at least 72 h before any experiments.

### Ephys recording

On the day of the electrophysiological (ephys) recording, a helium-filled mylar balloon was tied to the cables to alleviate the weight, enabling the mouse to move freely. All the *in vivo* ephys recordings were made with a Plexon MAP system (Plexon). The signals were filtered through the preamplifier to record neuronal spiking activities and local field potentials (LFPs) separately. The spike signals were filtered from 400 to 7000 Hz and sampled at 40 kHz, while the LFP signals were filtered from 0.7 to 300 Hz and sampled at 1 kHz. The optrode was advanced manually by ∼35 μm every 3 d before they reach stratum pyramidale of the dorsal CA1 area of the hippocampus. The position of the tetrode tips was estimated by observing apparent sharp wave-ripple events in the LFP signals during slow-wave sleep in the home cage. We began the recording after most of the tetrodes have reached stratum pyramidale and spontaneous firings of different neurons were observed. Animal behavior was simultaneously recorded along ephys data by a camera on top of the recording arena. After the ephys recording experiments, the animals were killed for histologic staining of brain slices with 1% cresyl violet to confirm the position of the optrode.

### Optogenetic stimulation

The optic fiber within the optrode was coupled to an external fiber using standard FC connectors via the ceramic sleeve and then connected to a Diode-Pumped Solid State (DPSS) Laser (589 nm, Inper). Laser power at the output end of the optic fiber was 10 mW/mm^2^. In each 30-min standard ephys recording session, two or three times of continues optogenetic stimulation was delivered, each lasting for 2 or 3 min. The optogenetic stimulation protocol was preprogramed with the DPSS Laser and the signal was synchronized with the ephys recording via a digital input cable connected to the Plexon MAP system.

### Spike sorting and data selection

**Figure 1.**
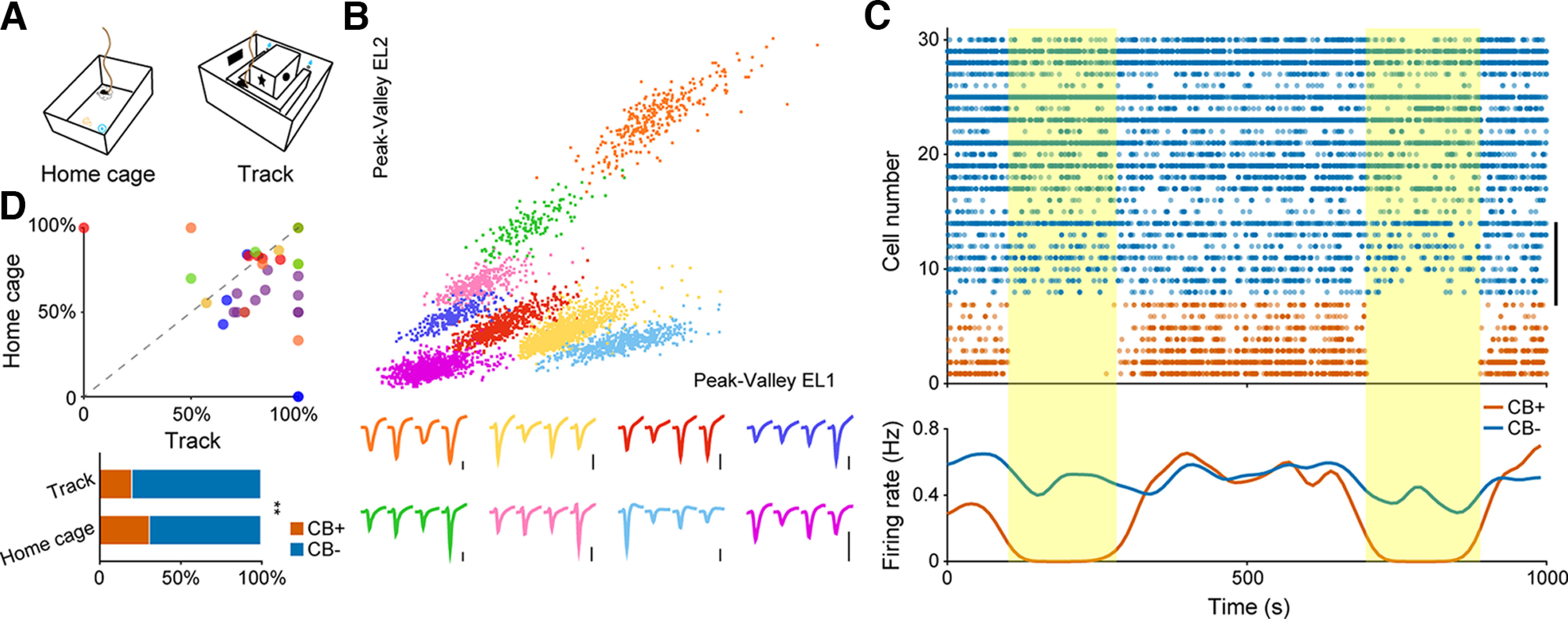
*In vivo* identification and basic firing pattern of hippocampal pyramidal neuron subtypes. ***A***, Two recording setups used in our experiment. Left, Home cage. Right, U-shape running track. ***B***, Eight putative pyramidal neurons sorted from a single tetrode. Each color denotes one single unit. Corresponding tetrode waveforms were illustrated at the bottom. The orange cluster in the upper right corner represents one CB+ neuron. Scale bar: 0.2 mV. ***C***, Neuronal firing sequence of 30 simultaneously recorded pyramidal neurons for a 1000-s recording period in home cage. Vertical bar on the right denotes eight neurons recorded from one single tetrode, as illustrated in ***B***. Each dot represents one action potential. The firing of CB+ PNs were largely inhibited on optogenetic stimulation (yellow shaded areas). Bottom, Average firing rate of these neurons showing population responses of PNs to laser stimulation. Blue, CB− PNs; orange, CB+ PNs. ***D***, Top, Ratio of CB− PNs recorded from each tetrode in running track as a function of that in home cage. Each dot represents result from one tetrode, while each color denotes individual animal (*n* = 6 mice). Bottom, Ratio of active CB+ and CB− PNs recorded from two recording setups. Note the significant decrease of the ratio of CB+ neurons recorded during running (**p = 6.6e-3, χ^2^ test).

All the ephys and external signals were stored in a single .plx file. Spike sorting was performed manually using Plexon Offline Sorter (version 2.7.3) in a 2D and sometimes 3D feature space. The degree to which the selected unit clusters were separated in the 2D or 3D space was determined by a multivariate ANOVA (MANOVA) test. The smaller the *p*-value, the more confident one can be of the conclusion that the clusters were in fact distinct and represent different units. Also, during the spike sorting process, sorting quality was closely monitored after each sorting operation by the following built-in statistics in Offline Sorter: J3 and Pseudo-F. For example, the majority of the neurons in Figure 1 were recorded from one tetrode. The MANOVA statistics were: *p* = 1.61e-77, *F* = 13.85 in a 2D cluster space, and *p* = 1.77e-117, *F* = 14.10 in a 3D cluster space. J3 statistics in a 2D and 3D space were 7.38 and 6.36. Pseudo-*F* statistics in a 2D and 3D space were 5442.2 and 4686.16.

CA1 pyramidal neurons and interneurons were identified based on their action potential waveforms and corresponding firing rates (typically <6 Hz for pyramidal neurons and >10 Hz for interneurons). A total number of 327 pyramidal neurons and six interneurons in the running track and 373 pyramidal neurons and 8 interneurons in home cage were sorted from six mice. Only pyramidal neurons were used in this study. Pyramidal neurons with an average firing rate below 0.01 Hz in the home cage recording are excluded from the dataset. Place cell selection criteria was described below.

### Identification of CB+ and CB− PNs

During each recording session, a brief yellow laser stimulation was delivered to inhibit neuronal firings of CB+ PNs. We calculated the laser inhibition index to identify CB+ PNs from the data pool. Laser inhibition index was calculated as follow:

$$\text{Inhibition index}=\left(1-\frac{Average\;firing\;rate_{during\;laser\;stimulation}}{Average\;firing\;rate_{non\;laser\;stimulation}}\right)*100\%.$$

A neuron was marked as CB+ whenever the neuronal laser inhibition index exceeds 80%. In our dataset, the total number of CB− PNs was considerably higher than CB+ PNs (number of recorded neurons reported in Fig. 1*D*). This was largely because of technical limitations of tetrode recordings. CB− PNs reside at the deep layer of stratum pyramidale (next to stratum Oriens), which was more accessible for our tetrode bundles. In order to maximize the total number of neurons recorded, we chose to start the recording session once the tetrode bundle had reached the stratum pyramidale, hence more deep layer pyramidal neurons, presumably CB− PNs, were recorded.

### Behavior states selection

We selected three behavior states for further firing pattern analysis of CB+ and CB− PNs: active running (RUN), REM sleep (REM) and slow wave sleep (SWS). An overhead camera recorded animal behavior simultaneously with ephys recording. RUN state was selected during active running in the track with a running speed over 5 cm/s. Behavior video recordings were used to identify sleep states, which were further divided into REM and SWS states based on distinct local field potential patterns of theta and ripple oscillations.

### Data analysis

#### Burst analysis

We used the MaxInterval method to detect burst events (NeuroExplorer, Nex Technologies). A burst should at least contain two consecutive spikes. For bursts with more than two spikes, the interval between the first two spikes should be <10 ms, while the interval between any two consecutive spikes should be <20 ms. The duration of a burst should be at least >3 ms (two spikes minimum). The interburst interval should be >20 ms. Burst frequency is defined as the number of bursts over a time range divided by that duration. Burst index was defined as the total number of bursting spikes divided by the number of overall spikes committed by that neuron.

#### Detection of theta and ripple oscillations

We used the same method reported in our previous report to detect different oscillation patterns (Ma et al., 2020). In brief, the original LFP was bandpass filtered (4–12 Hz) for the detection of theta oscillations. Theta epochs were detected by calculating the power ratio of the theta (5–10 Hz) and δ (2–4 Hz) band by sliding a 2-s window. Epochs with more than three consecutive time windows in which the ratio was >4 were identified as theta episodes (Csicsvari et al., 1999).

To detect ripple events, the original LFP was first bandpass filtered (100–250 Hz). The power of the filtered signal was calculated by sliding a 10-ms window every 1 ms. The threshold for ripple detection was set to 5 SD above the background mean power. The beginning and end of each ripple epoch were identified by sliding the time window forward and backward, by the threshold of 2 SD above the background mean power (Csicsvari et al., 1999).

#### Phase analysis

The theta or ripple bandpass filtered LFP was first decomposed into instantaneous amplitude ρ(t) and phase ϕ(t) components by using a Hilbert transform:

$$y(t)=Re(\rho (t)e^{j\phi (t)}).$$

Given the neuronal spike train $\left\{t_{i}\text{| }i=1,2,...,n\right\}$, spike phase was calculated by

$$\varphi_{i}=\varphi \left(t_{i}\right).$$

We define oscillation peaks at 0° and 360° and troughs at 180° throughout the paper. The mean direction and mean resultant length of the phases of a given neuron’s spikes were taken as the preferred firing phase and modulation depth of that neuron, respectively. To evaluate the presence of phase locking, we performed Rayleigh’s test for circular uniformity to compute the significance of phase locked firing. Only neurons that showed an average firing rate over 0.1 Hz during theta or ripple epochs, and significantly modulated by the oscillations (Rayleigh’s test, *p* < 0.05) were included in the analyses. To avoid theta/ripple phase variability as a function of recording depth, the electrode with the largest ripple power (that is, the middle of the CA1 pyramidal layer) was used as reference for detecting theta/ripple phase.

#### Ripple participation and inhibition index

For each neuron, the fraction of ripples with neuronal spike(s) was calculated as ripple participation to represent the extent of neuronal involvement in ripple oscillations. The ripple inhibition index was defined as follow to describe the degree of inhibition of neuronal firing after ripple peak:

$$Inhibition\;index=\left(1-\frac{Post-ripple\;FR}{Baseline\;FR}\right)*100\%.$$

Where postripple firing rate (FR) was the average firing rate for the 100 ms after ripple peak, baseline FR was the baseline neuronal firing rate excluding the 100-ms period before and after ripple peak.

#### Place cell analysis

A total number of 327 pyramidal neurons fitted the criteria for data selection from 6 mice in the track running experiments (66 CB+ PNs and 261 CB− PNs). To characterize place fields of CA1 pyramidal neurons, we first linearized and then binned the U-shape track with 1 cm width bin. Average firing rate of each running direction (clockwise and counterclockwise) was calculated by dividing the total number of spikes in each bin by the occupancy time in that bin. The place field of a neuron was the bins where the neuron displayed the highest firing rate and all contiguous bins exceeded 20% of peak firing rate. Neurons that showed a peak firing rate over 2 Hz with a clear place field preference were defined as place cells. Furthermore, cells with a place field covering more than half of the track were excluded from the analysis because of low place specificity. Following these criteria, we identified 27 CB+ and 163 CB− place cells from the data pool.

#### Spatial information

Spatial information content was calculated in bits per second (I_second_) using the following formula:

$$I_{second}=\overset{N}{\underset{i=1}{\sum }}p_{i}\lambda_{i}log_{2}\frac{\lambda_{i}}{\lambda }.$$

Where *N* was the number of bins of the track, $\lambda_{i}$ was the mean firing rate of a neuron in the i-th bin, λ was the overall mean firing rate of the neuron, $p_{i}$ was the probability that animal being in the i-th bin (occupancy in the i-th bin divided by the total recording time).

Spatial information content can also be calculated in bits per spike (I_spike_) when divided by the overall firing rate of the neuron:

$$I_{spike}=\overset{N}{\underset{i=1}{\sum }}p_{i}\frac{\lambda_{i}}{\lambda }log_{2}\frac{\lambda_{i}}{\lambda }.$$

#### Sparsity and selectivity

Place cell firing sparsity measures the fraction of the environment in which a neuron was active. The sparsity index was calculated following Skaggs (Skaggs et al., 1996) as:

$$Sparsity=\frac{{\left(\sum_{i}p_{i}\lambda_{i}\right)}^{2}}{\sum_{i}p_{i}\lambda_{i}^{2}}.$$

Spatial firing selectivity measures how concentrated the neuron’s activity was. Neurons with no spatial tuning will have a selectivity of 1. Higher selectivity index represents more tightly concentrated place cell firing. Selectivity index was defined as:

$$Selectivity=\frac{Maximum\;firing\;rate}{Average\;firing\;rate}.$$

#### Phase precession

We used a circular-linear regression method (Maier et al., 2011) to quantify properties of theta phase precession of place cells. Phase precession was calculated within each place field of place cells. Linearized track was binned into 1-cm width bin and neuronal firing rate was calculated for each bin. The place field of a neuron was the bins where the neuron displayed the highest firing rate and all contiguous bins exceeded 20% of peak firing rate. Place field was normalized so that 0 represented the beginning and 1 represented the end of each place field. Phase precession was defined as significant negative linear-circular correlation (*p* < 0.05, linear-circular correlation test) between the animal position in the normalized place field and the theta firing phase (Berens, 2009).

#### Quantification and statistical analysis

All statistical analyses were performed in MATLAB (MathWorks). Data were presented as mean ± SEM unless stated otherwise. Shapiro–Wilk test and Kolmogorov–Smirnov test were used to determine whether sample distribution was standard normal distribution. If normality was uncertain, nonparametric tests were used. Details of the statistical tests and the resultant *p*-values were listed in the main text and figure legends. In each box-plot figure, the central mark indicates the median, and the bottom and top edges of the box indicate the 25th and 75th percentiles, respectively. The whiskers extend to the most extreme data points not considered outliers. Outlier values were always included in the statistical analysis, although they were not represented in the plots. Circular statistics toolbox for MATLAB was used for comparison in polar coordinates (Berens, 2009). Rayleigh’s test was applied to check whether population is uniformly distributed around the circle. Statistical details are provided in Table 1.

## Results

### Basic firing patterns of hippocampal CB+ and CB− PN subtypes under different behavior states

**Figure 2.**
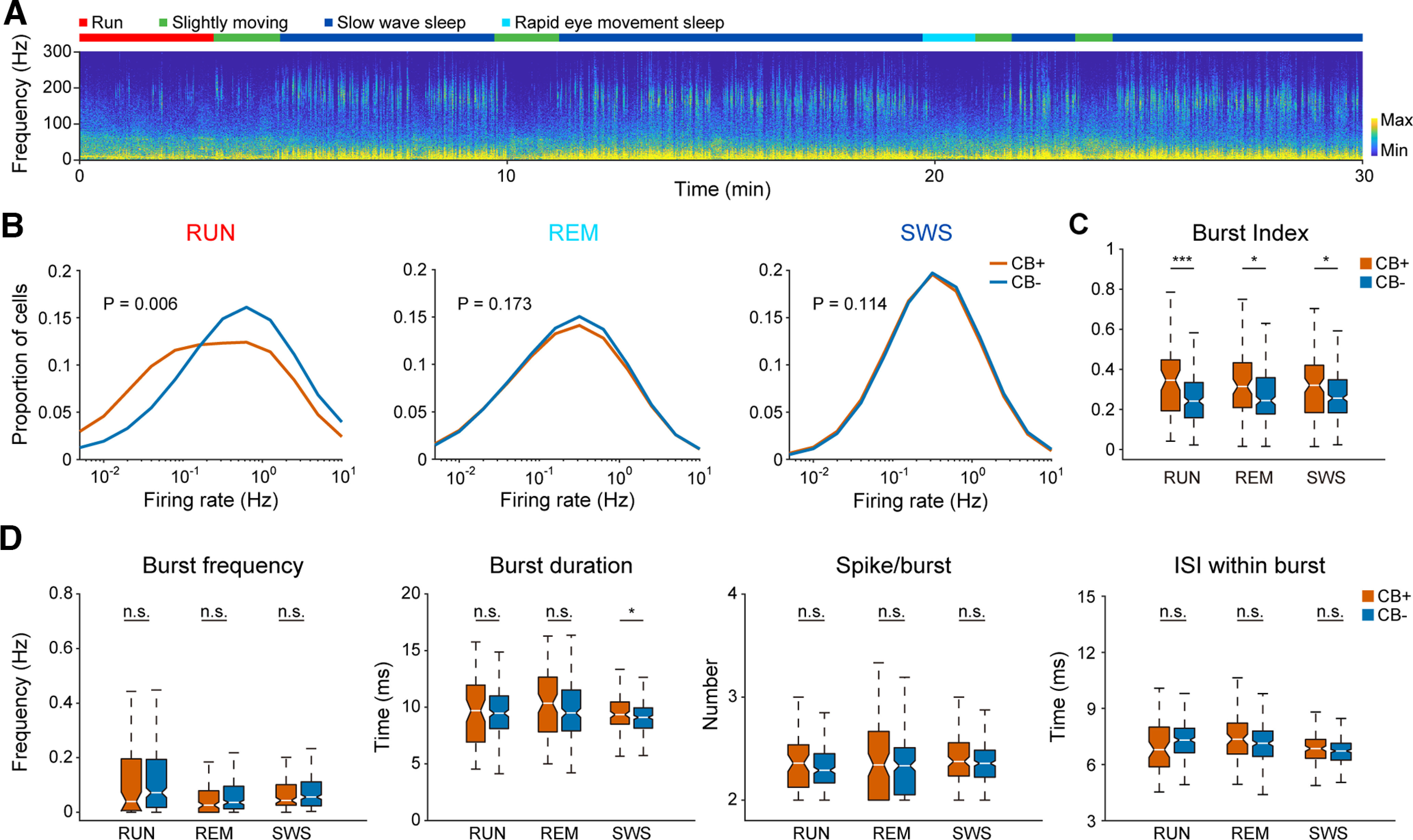
*In vivo* identification and basic firing pattern of hippocampal pyramidal neuron subtypes. ***A***, Spectrogram of hippocampal CA1 local field potential during a 30-min home cage recording session. Different behavior states are indicated by different colors at the top. Note the high oscillation power in ripple band during SWS and the absence of ripple power during theta states (i.e., RUN and REM sleep). ***B***, Average firing rate of CB+ and CB− PNs during three behavior states: RUN, REM, and SWS. CB− PNs showed higher firing rate than CB+ PNs during RUN state (*n* = 66 for CB+ PNs and 261 for CB− PNs, *p* = 6e-3, Mann–Whitney test), but not during REM and SWS states (*n* = 116 for CB+ PNs and 257 for CB− PNs, REM, *p* = 0.173; SWS, *p* = 0.114, Mann–Whitney test). ***C***, Burst index of CB+ PNs were significantly higher than that of CB− PNs under all three behavior states (neuron number is same as in ***B***; RUN, ***p = 5e-4; REM, *p = 0.02; SWS, *p = 0.031, Mann–Whitney test). ***D***, Burst firing parameters of both PN subtypes under different behavior states, including burst frequency, duration, number of spikes and interspike interval within bursts (neuron number is same as in ***B***, n.s., not significant; *p = 0.029, Mann–Whitney test).

We performed multichannel *in vivo* ephys recordings in 10 transgenic mice that had Arch-GFP selectively expressed in CB+ PNs. Ephys data were collected in two behavior setups: home cage and a U-shaped track (Fig. 1*A*). Well-isolated pyramidal neurons with an average firing rate over 0.01 Hz were selected for further analysis (Fig. 1*B*). During each recording session, a brief yellow laser stimulation of 3 min was delivered to inhibit the neuronal firing of CB+ PNs. Based on the firing inhibition profile during light stimulation, we categorized all the sorted pyramidal neurons as CB+ and CB− PNs (Fig. 1*C*). We collected a total of 116 CB+ and 257 CB− PNs in home cage recording, 66 CB+ and 261 CB− PNs in the running track. The number of CB− PNs recorded from each tetrode was higher than CB+ PNs (Fig. 1*D*; also, for detailed explanation, see Materials and Methods). We selected three distinct behavioral states for further analysis, RUN, SWS, and REM sleep, based on local field potential (LFP) patterns (Fig. 2*A*) and simultaneously recorded animal behavior videos.

We found that the firing rate of CB− neurons were significantly higher than that of CB+ PNs during active running, but not during sleep states (Table 2; Fig. 2*B*). Although the two PN subtypes showed similar kinetics of burst firings (Fig. 2*D*, including burst frequency, duration, number of spikes and ISI within burst), CB+ PNs showed a higher burst index than CB− PNs under all three behavior states (Table 2; Fig. 2*C*). Note that CB+ PNs showed a longer burst duration than that of CB− PNs during SWS (Fig. 2*D*).

**Table 1:** Statistical table

Figure	Data structure	Type of test	Statistic report
1*D*, bottom		χ^2^ test	c^2^ (1, *n* = 700) = 10.79, *p* = 0.0066
2*B*	Non-normal distribution	Mann–Whitney test	Mann–Whitney *U* = 6117, *n*1 = 62, *n*2 = 255, *p*_RUN_ = 0.0057, two-tailed
			Mann–Whitney *U* = 137,446, *n*1 = 341, *n*2 = 849, *p*_REM_ = 0.1725, two-tailed
			Mann–Whitney *U* = 930,283, *n*1 = 941, *n*2 = 2051, *p*_SWS_ = 0.1136, two-tailed
2*C*	Non-normal distribution	Mann–Whitney test	Mann–Whitney *U* = 5195, *n*1 = 64, *n*2 = 226, *p*_RUN_ = 0.0005, two-tailed
			Mann–Whitney *U* = 7470, *n*1 = 88, *n*2 = 205, *p*_REM_ = 0.0195, two-tailed
			Mann–Whitney *U* = 13,197, *n*1 = 121, *n*2 = 253, *p*_SWS_ = 0.031, two-tailed
2*D*, burst frequency	Non-normal distribution	Mann–Whitney test	Mann–Whitney *U* = 8071, *n*1 = 73, *n*2 = 255, *p*_RUN_ = 0.0832, two-tailed
			Mann–Whitney *U* = 13,775, *n*1 = 122, *n*2 = 253, *p*_REM_ = 0.0901, two-tailed
			Mann–Whitney *U* = 14,877, *n*1 = 122, *n*2 = 253, *p*_SWS_ = 0.5722, two-tailed
2*D*, burst duration	Non-normal distribution	Mann–Whitney test	Mann–Whitney *U* = 7226, *n*1 = 64, *n*2 = 226, *p*_RUN_ = 0.9916, two-tailed
			Mann–Whitney *U* = 7882, *n*1 = 88, *n*2 = 205, *p*_REM_ = 0.087, two-tailed
			Mann–Whitney *U* = 13,165, *n*1 = 121, *n*2 = 253, *p*_SWS_ = 0.0286, two-tailed
2*D*, spike/burst	Non-normal distribution	Mann–Whitney test	Mann–Whitney *U* = 6614, *n*1 = 64, *n*2 = 226, *p*_RUN_ = 0.2972, two-tailed
			Mann–Whitney *U* = 8480, *n*1 = 88, *n*2 = 205, *p*_REM_ = 0.413, two-tailed
			Mann–Whitney *U* = 14,194, *n*1 = 121, *n*2 = 253, *p*_SWS_ = 0.2553, two-tailed
2*D*, ISI within burst	Non-normal distribution	Mann–Whitney test	Mann–Whitney *U* = 6109, *n*1 = 64, *n*2 = 226, *p*_RUN_ = 0.0578, two-tailed
			Mann–Whitney *U* = 7974, *n*1 = 88, *n*2 = 205, *p*_REM_ = 0.1157, two-tailed
			Mann–Whitney *U* = 13,397, *n*1 = 121, *n*2 = 253, *p*_SWS_ = 0.0509, two-tailed
3*D*		χ^2^ test	c^2^ (1, *n* = 327) = 10.04, *p* = 0.0015
3*E*	Non-normal distribution	Mann–Whitney test	Mann–Whitney *U* = 1967, *n*1 = 27, *n*2 = 163, *p* = 0.3588, two-tailed
3*F*		χ^2^ test	c^2^ (2) = 7.102, *p* = 0.0287
3*G*	Non-normal distribution	Mann–Whitney test	Mann–Whitney *U* = 6424, *n*1 = 54, *n*2 = 367, *p* = 0.7982, two-tailed
3*H*, left	Non-normal distribution	Mann–Whitney test	Mann–Whitney *U* = 9489, *n*1 = 54, *n*2 = 367, *p* = 0.6153, two-tailed
3*H*, right	Non-normal distribution	Mann–Whitney test	Mann–Whitney *U* = 9478, *n*1 = 54, *n*2 = 367, *p* = 0.6061, two-tailed
3*I*	Non-normal distribution	Mann–Whitney test	Mann–Whitney *U* = 7174, *n*1 = 54, *n*2 = 367, *p* = 0.001, two-tailed
3*J*, left	Non-normal distribution	Mann–Whitney test	Mann–Whitney *U* = 6835, *n*1 = 54, *n*2 = 326, *p* = 0.0085, two-tailed
3*J*, right	Non-normal distribution	Mann–Whitney test	Mann–Whitney *U* = 8144, *n*1 = 54, *n*2 = 326, *p* = 0.3791, two-tailed
3*K*	Non-normal distribution	Mann–Whitney test	Mann–Whitney *U* = 6709, *n*1 = 54, *n*2 = 326, p_bits/spk_ = 0.0051, two-tailed
			Mann–Whitney *U* = 7850, *n*1 = 54, *n*2 = 326, p_bits/s_ = 0.2031, two-tailed
3*N*, left	Non-normal distribution	Mann–Whitney test	Mann–Whitney *U* = 2710, *n*1 = 32, *n*2 = 171, *p* = 0.9337, two-tailed
3*N*, middle	Non-normal distribution	Mann–Whitney test	Mann–Whitney *U* = 2667, *n*1 = 32, *n*2 = 171, *p* = 0.8231, two-tailed
3*N*, right	Non-normal distribution	Mann–Whitney test	Mann–Whitney *U* = 2649, *n*1 = 32, *n*2 = 171, *p* = 0.7777, two-tailed
4*C*, left		Watson’s U^2^ test	*p* = 0.0002
4*C*, right	Non-normal distribution	Mann–Whitney test	Mann–Whitney *U* = 3190, *n*1 = 43, *n*2 = 199, *p* = 0.0086, two-tailed
4*D*, left		Watson’s U^2^ test	*p* = 0.0990
4*D*, right	Non-normal distribution	Mann–Whitney test	Mann–Whitney *U* = 3346, *n*1 = 52, *n*2 = 181, *p* = 0.0014, two-tailed
4*E*, left		Watson’s U^2^ test	*p* = 0.0091
4*E*, right	Non-normal distribution	Mann–Whitney test	Mann–Whitney *U* = 370, *n*1 = 52, *n*2 = 43, *p* = 2.28E-08, two-tailed
4*F*, left		Watson’s U^2^ test	*p* = 0.4460
4*F*, right	Non-normal distribution	Mann–Whitney test	Mann–Whitney *U* = 6616, *n*1 = 181, *n*2 = 199, *p* = 1.68E-26, two-tailed
5*D*	Non-normal distribution	Mann–Whitney test	Mann–Whitney *U* = 9242, *n*1 = 116, *n*2 = 257, *p* = 4.21E-09, two-tailed
5*E*	Non-normal distribution	Mann–Whitney test	Mann–Whitney *U* = 9071, *n*1 = 116, *n*2 = 257, *p* = 1.42E-09, two-tailed
5*F*	Non-normal distribution	Mann–Whitney test	Mann–Whitney *U* = 7329, *n*1 = 116, *n*2 = 257, *p* = 3.84E-15, two-tailed
5*H*, left	Non-normal distribution	Mann–Whitney test	Mann–Whitney *U* = 11,320, *n*1 = 116, *n*2 = 257, *p* = 0.0013, two-tailed
5*H*, right	Non-normal distribution	Mann–Whitney test	Mann–Whitney *U* = 10,692, *n*1 = 116, *n*2 = 257, *p* = 1.2E-5, two-tailed
5*I*, left	Non-normal distribution	Mann–Whitney test	Mann–Whitney *U* = 10,538, *n*1 = 116, *n*2 = 257, *p* = 5.9E-6, two-tailed
5*I*, right	Non-normal distribution	Mann–Whitney test	Mann–Whitney *U* = 8397, *n*1 = 116, *n*2 = 257, *p* = 1.5E-11, two-tailed
6*C*		Watson’s U^2^ test	*p* = 0.124
6*D*	Non-normal distribution	Mann–Whitney test	Mann–Whitney *U* = 3922, *n*1 = 60, *n*2 = 162, *p* = 0.027, two-tailed

**Table 2:** Firing pattern statistics of CB+ and CB− PNs (related to **Figs. 1, 3, and 5).**

	CB+ (mean ± SEM)	CB− (mean ± SEM)
Average firing rate_RUN (Hz)	0.931 ± 0.154	1.322 ± 0.092
Average firing rate_REM (Hz)	0.558 ± 0.040	0.584 ± 0.027
Average firing rate_SWS (Hz)	0.632 ± 0.018	0.674 ± 0.014
Burst index_RUN	0.337 ± 0.022	0.255 ± 0.009
Burst index_REM	0.325 ± 0.019	0.282 ± 0.011
Burst index_SWS	0.313 ± 0.015	0.28 ± 0.009
Place field size (cm)	29.81 ± 1.474	31.11 ± 0.829
In field aFR (Hz)	5.793 ± 0.623	5.344 ± 0.215
In field pFR (Hz)	9.029 ± 0.948	8.372 ± 0.332
In/out field FR	17.63 ± 1.606	13.24 ± 0.607
Sparsity	0.177 ± 0.026	0.229 ± 0.01
Selectivity	11.17 ± 0.816	10.8 ± 0.378
Spatial information (bits/spk)	2.338 ± 0.182	1.809 ± 0.064
Spatial information (bits/s)	1.253 ± 0.146	1.114 ± 0.058
aFR in ripple (Hz)	0.659 ± 0.059	1.245 ± 0.07
pFR in ripple (Hz)	1.66 ± 0.121	2.429 ± 0.119
FR in/out ripple	1.028 ± 0.058	1.707 ± 0.053
Ripple participation	0.038 ± 0.003	0.067 ± 0.003
Peak time (ms)	−19.74 ± 4.149	−11.28 ± 2.062
Baseline FR (Hz)	0.643 ± 0.046	0.775 ± 0.039
Postripple FR (Hz)	0.515 ± 0.039	0.798 ± 0.042
Inhibition index	0.19 ± 0.037	−0.087 ± 0.027

**Figure 3.**
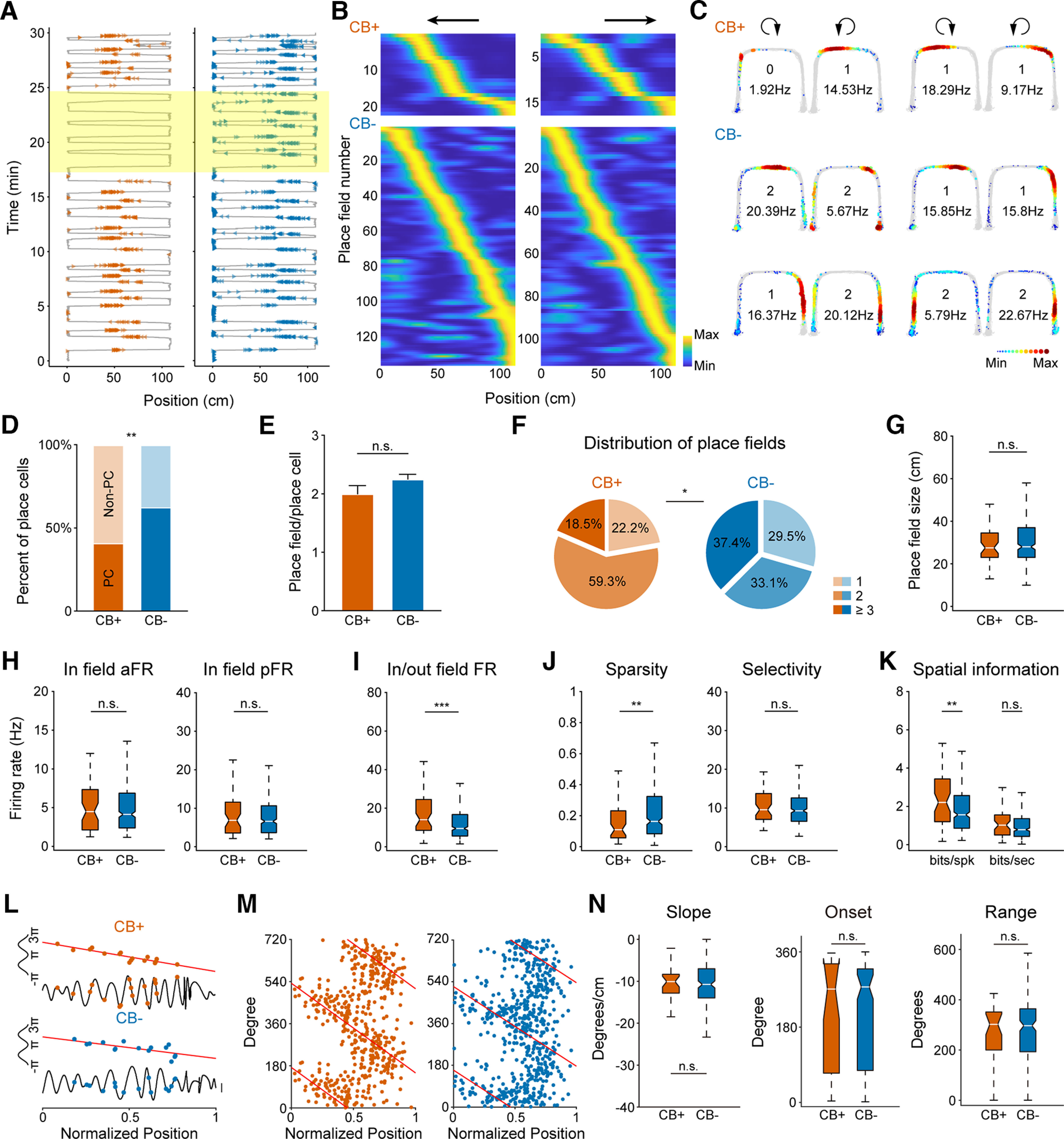
CB+ place cells represent spatial information more efficiently than CB− place cells. ***A***, Two example place cell firings along the linearized U-shape track. Left, CB+ place cell. Right, CB− place cell. Gray lines depict animal running trajectory. Colored triangles are spikes from each place cell. Place-selective firing of CB+ neuron is suppressed on optogenetic inhibition (yellow shaded box). ***B***, Population activities of both CB+ and CB− place cells distributed along the running track on both directions. Neuronal firings are normalized and sorted by their relative peak firing position along the track (*n* = 27 for CB+ and 163 for CB− place cells). ***C***, Example place cells recorded from one mouse. Clockwise and counter-clockwise runnings are separated and indicated by a circular arrow at the top. Gray lines are overlapped animal running trajectories. Colored dots represent firing rate of each place cell. Number of place fields and peak firing rate are shown in the middle of each figure. ***D***, Place cell ratio of CB+ PNs is significantly lower than that of CB− PNs (CB+: *n* = 27 place cells and 39 nonplace cells, CB−: *n* = 163 place cells and 98 nonplace cells, **p = 2e-3, χ^2^ test). ***E***, Number of place fields formed by CB+ and CB− place cells (n.s., not significant, *p* = 0.359, Mann–Whitney test). ***F***, Distribution of varying number of place fields of CB+ and CB− place cells, with one, two, or more than three place fields (*p = 0.029, χ^2^ test). ***G***, Place field size of both CB+ and CB− place cells (n.s., not significant, *p* = 0.798, Mann–Whitney test). ***H***, Average firing rate (aFR) and peak firing rate (pFR) within place fields (n.s., not significant, *p* = 0.615 for aFR, 0.606 for pFR, Mann–Whitney test). ***I***, Firing rate ratio inside and outside place fields. CB+ place cells fire significantly more spikes inside than outside the place fields than that of CB− place cells (***p = 1e-3, Mann–Whitney test). ***J***, Neuronal firing sparsity and selectivity of place cells (**p = 9e-3; n.s., not significant, *p* = 0.379, Mann–Whitney test). ***K***, Spatial information calculated by every spike or second of both place cell subtypes. CB+ place cells carry more information per spike than CB− place cells (**p = 5e-3; n.s., not significant, *p* = 0.203, Mann–Whitney test). ***L***, Example phase precession of both place cell subtypes during one place field traverse. Two normalized theta cycles are shown for clarity. ***M***, Phase precession of CB+ and CB− place cells. Two theta cycles are shown. ***N***, No significant difference found in phase precession parameters, including slope, onset, and range (*p* = 0.934, *p* = 0.823, *p* = 0.778, Mann–Whitney test).

**Figure 5.**
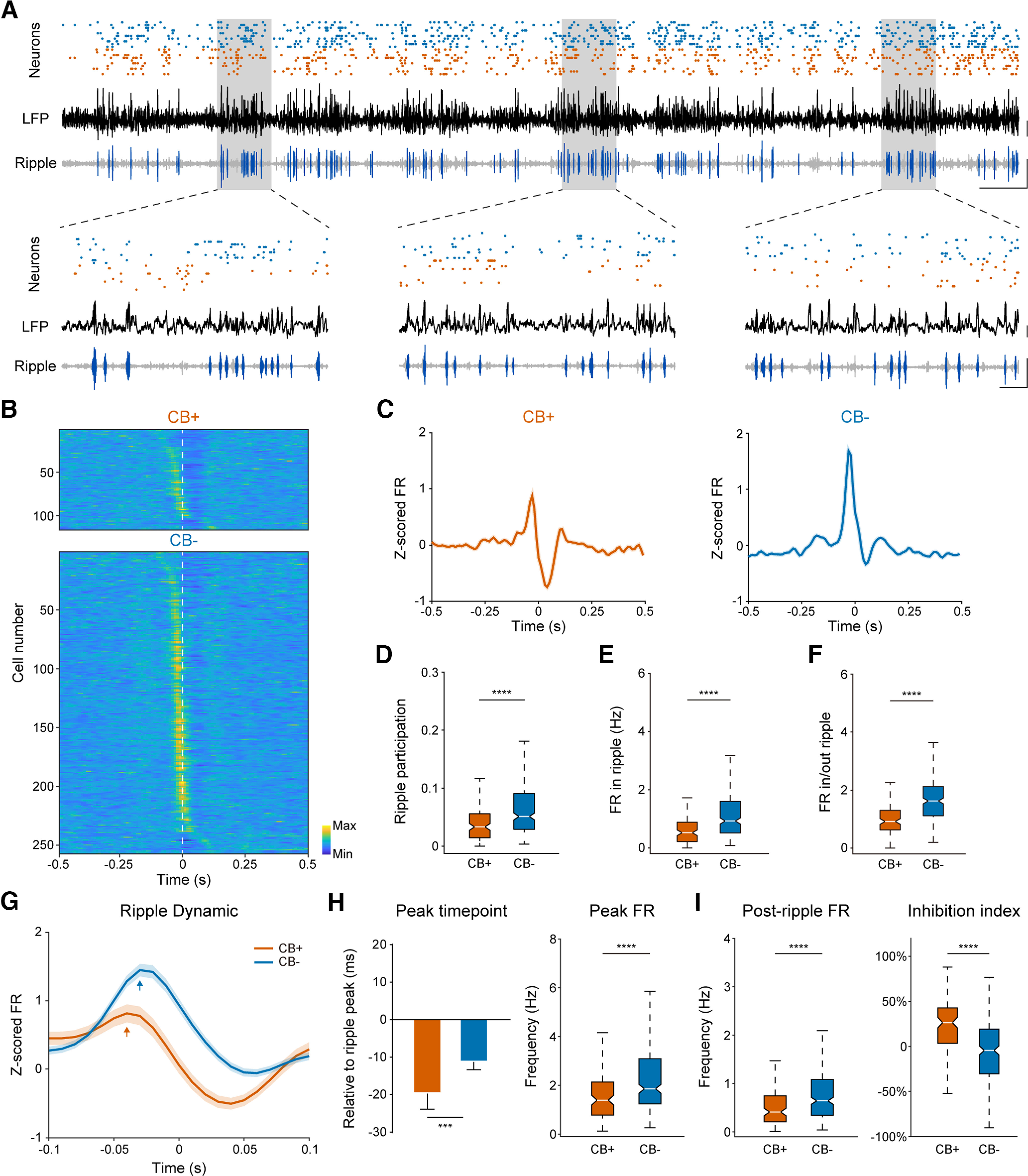
CB− PNs are more actively engaged in hippocampal ripple oscillations during SWS. ***A***, Top, Neuronal firing sequence of ten CB+ (blue dots) and ten CB− (orange dots) PNs along with hippocampal LFP (black trace) and bandpass filtered ripple oscillation (gray trace with ripple events highlighted in dark blue) during SWS. Scale bar: 0.2 mV, 0.5 s. ***B***, Normalized neuronal firing of both PN subtypes during ripple oscillations (*n* = 3888 ripple events), sorted by peak firing time (*n* = 116 CB+ PNs, and 257 CB− PNs). Time zero represents ripple peak (white broken line). Note that CB− PNs exhibited higher firing rate before ripple peak, while CB+ PNs showed lower firing rate after LFP ripple peak. ***C***, Normalized firing rate of each PN subtype population during hippocampal ripple oscillations. Time zero denotes ripple peak. Note that population activity of CB+ PNs are briefly suppressed after ripple peak. Bin size: 10 ms. ***D***, Ripple participation of both PN subtypes. CB− PNs are more actively engaged in ripple oscillations (****p = 4.2e-9, Mann–Whitney test). ***E***, Average firing rate of both neuron subtypes during ripple oscillations (****p = 1.4e-9, Mann–Whitney test). ***F***, Firing rate ratio inside and outside ripple oscillations of both CB+ and CB− PNs (****p = 3.8e-15, Mann–Whitney test). ***G***, Normalized firing rate dynamics of both PN subtypes during ripples. Shown here is 0.1-s data around ripple peak. Bin size: 10 ms. ***H***, Time and peak firing rate of both PN subtypes relative to LFP ripple peak. CB+ PNs participated earlier in ripples than CB− PNs (***p = 1.3e-3, ****p = 1.2e-5, Mann–Whitney test). ***I***, Left, Postripple firing rate of both PN subtypes. The activity of CB+ PNs decreased after ripple, and also significantly lower than CB− PNs (****p = 5.9e-6, Mann–Whitney test). Right, Ripple inhibition index of both PN subtypes. CB+ PNs were more strongly inhibited after ripple peak than their CB− peers (****p = 1.5e-11, Mann–Whitney test).

### Efficient representation of spatial information by CB+ place cells

Since the discovery of place cells in rat hippocampus, neuronal representation of spatial information is one of the most fundamental and widely studied function of the hippocampus (O’Keefe and Dostrovsky, 1971; Moser et al., 2017). The higher firing rate of CB− PNs during running led us to speculate potential functional difference in spatial representation. To test this, we recorded place cell activities while the mice was trained to shuttle back and forth on a U-shape track. Yellow light stimulation was delivered during running on the track to inhibit neuronal firing of CB+, but not CB− PNs (Fig. 3*A*). We collected a total of 66 CB+ and 261 CB− PNs from 6 mice during track running. Among the data pool, we identified 27 CB+ and 163 CB− place cells, with 54 and 367 place fields for each place cell subtype (for data selection criteria, see Materials and Methods). Place cell activities of both CB+ and CB− PNs can cover the whole track (Fig. 3*B*), while place fields formed on each running direction were slightly different for both PN subtypes (Fig. 3*C*, two CB+ and four CB− PNs on each running direction are illustrated). CB+ PNs were less likely to be place cells than their negative peers, with only 40.9% of the recorded CB+ PNs are place cells, while 62.5% of the CB− PNs are place cells (Fig. 3*D*). The average number and size of place fields formed by each place cell subtypes are comparable (Table 2; Fig. 3*E*,*G*). Moreover, CB+ place cells were more likely to form double place fields (typically one on each running direction) than CB− place cells (Fig. 3*F*).

We next compared the spatial firing patterns of both place cell subtypes. Although they showed no difference in average and peak firing rate within place fields (Table 2; Fig. 3*H*), CB+ place cells exhibited higher firing rate inside than outside of their place fields compared with that of CB− place cells (Table 2; Fig. 3*I*). Consistently, the activity of CB+ place cells were more refined on the track, with higher spatial information per spike than CB− place cells (Table 2; Fig. 3*J*,*K*). Furthermore, the two place cell subtypes showed no difference in all aspects of phase precession characteristics (Fig. 3*L–N*).

Overall, we found that CB+ place cells encode spatial information more efficiently, as they fire in a more condensed manner inside and outside of place fields, with higher spatial information carried by each spike than that of CB− place cells.

### A subset of CB+ PNs shifted theta firing phase during REM sleep but not during RUN

**Figure 4.**
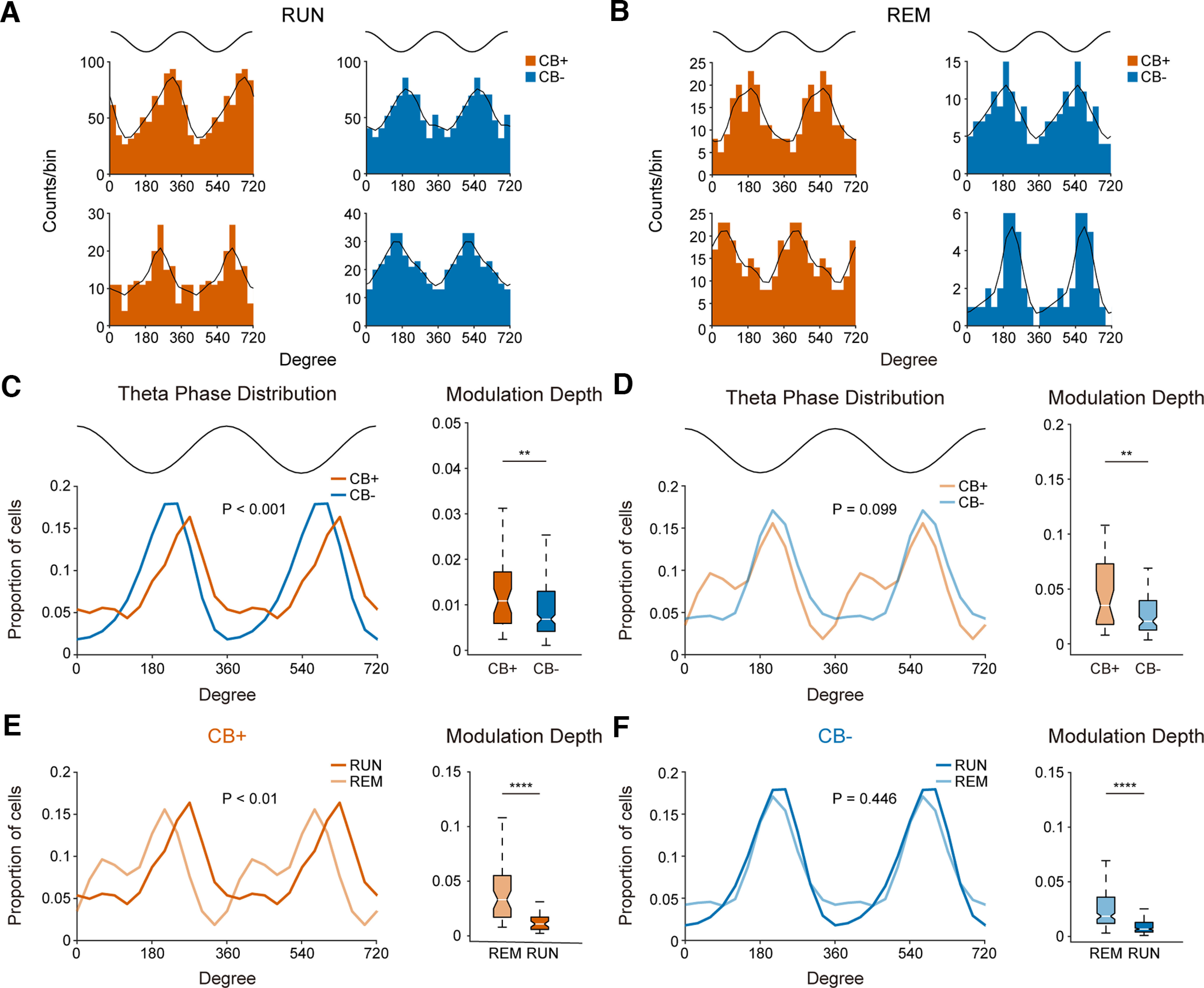
A subset of CB+ PNs shifted their theta firing phase during REM sleep. ***A***, Example theta firing phase distribution of both PN subtypes during RUN state (theta peak = 0°, 360°, theta trough = 180°; bin size: 30°). ***B***, Same as in ***A***, but for preferred theta firing phase during REM sleep theta oscillations. ***C***, Left, Distribution of preferred theta phase of both PN subtypes during RUN state (CB+ PNs, *n* = 43; CB− PNs, *n* = 199; *p* = 2.2e-4, Watson’s U^2^ test; bin size: 30°). Top trace indicates idealized reference theta cycle. Right, Theta modulation depth of CB+ and CB− PNs during RUN (**p = 8.6e-3 Mann–Whitney test). ***D***, Same as in ***C***, but for population distribution of preferred theta firing phase during REM sleep. Note the bimodal distribution of theta phase preference of CB+ PNs (*p* = 0.099, Watson’s U^2^ test; bin size: 30°). Right, Theta modulation depth of both PN subtypes during REM sleep (**p = 1.4e-3, Mann–Whitney test). ***E***, Left, Comparison of preferred theta firing phase of CB+ PNs during RUN and REM states. The preferred theta firing phase shifted significantly between the two states (*p* = 9.1e-3, Watson’s U^2^ test, bin size: 30°). Right, Modulation depth of CB+ PNs during REM sleep theta is significantly deeper than RUN theta states (****p = 2.3e-8, Mann–Whitney test). ***F***, Same as in ***E***, but for comparison of preferred theta firing phase of CB− PNs during RUN and REM states. No significant theta phase shift was observed between the two theta states (*p* = 0.446, Watson’s U^2^ test, bin size: 30°). Right, Theta modulation depth of CB− PNs during RUN and REM states (****p = 1.7e-26, Mann–Whitney test).

Previous study has reported that during REM sleep theta oscillation, a subset of hippocampal pyramidal neurons shifted their preferred theta firing phase, compared with that of running thetas (Mizuseki et al., 2011). We wondered whether it holds true for pyramidal neurons with distinct Calbindin expression profile. To do this, we calculated theta firing phase for each neuron during RUN and REM thetas (Fig. 4*A*,*B*). We found that firing activities of both PN subtypes are phase-locked to the ascending phase (180–360°) of theta oscillation during RUN (Fig. 4*A*,*C*), but they differ significantly in preferred theta phase (Fig. 4*C*).

During REM sleep theta oscillations, while the firing of most CB− PNs remained phase-locked to the ascending phase of theta oscillations as during RUN states, CB+ PNs shifted their theta firing phase dramatically (Fig. 4*B*,*D*). To determine the degree of theta phase shift, we compared the theta phase distribution of each PN subtypes during RUN and REM sleep. We found that the theta phase of CB+ PNs shifted significantly between RUN and REM sleep (Fig. 4*E*), while no such theta phase shift was observed in CB− PNs (Fig. 4*F*).

We also calculated the strength of theta phase-locking of both PN subtypes during the two theta states. CB+ PNs were more strongly phase-locked to theta oscillations than CB− PNs during both RUN and REM sleep (Fig. 4*C*,*D*). Furthermore, both PN subtypes were more deeply phase-locked to REM sleep theta than RUN theta oscillations (Fig. 4*E*,*F*).

### CB+ PNs are more strongly modulated by ripple, despite lower engagement during SWS

Activity of hippocampal pyramidal neurons during slow wave sleep, especially ripple oscillations is critical for hippocampal function of learning and memory (Girardeau et al., 2009; Buzsáki, 2015). So we looked into neuronal firing dynamics of both PN subtypes during ripple oscillations. We found that both PN subtypes increased their firing when ripple oscillations were prominent (Fig. 5*A*), but they were differentially engaged during ripples. First of all, CB− PNs were more actively engaged in ripples (Fig. 5*B*,*C*), as indicated by stronger participation in ripples, higher firing rate during ripples, and higher firing rate ratio inside and outside ripples of CB− PNs (Table 2; Fig. 5*D–F*). Such elevated activity of CB− PNs during ripples was not because of difference in baseline firing rate since the average firing rate of both PN subtypes are similar during SWS (Fig. 2*B*). It is also not caused by burst spikes since the deletion of burst spike did not change such elevated activity of CB− PNs (data not shown).

Second, CB+ PNs participated earlier in ripples than CB− PNs (Table 2; Fig. 5*H*). They were also more strongly suppressed after ripple peak as indicated by significantly lower postripple firing rate and higher ripple inhibition index of CB+ PNs than that of CB− PNs (Table 2; Fig. 5*B*,*I*).

**Figure 6.**
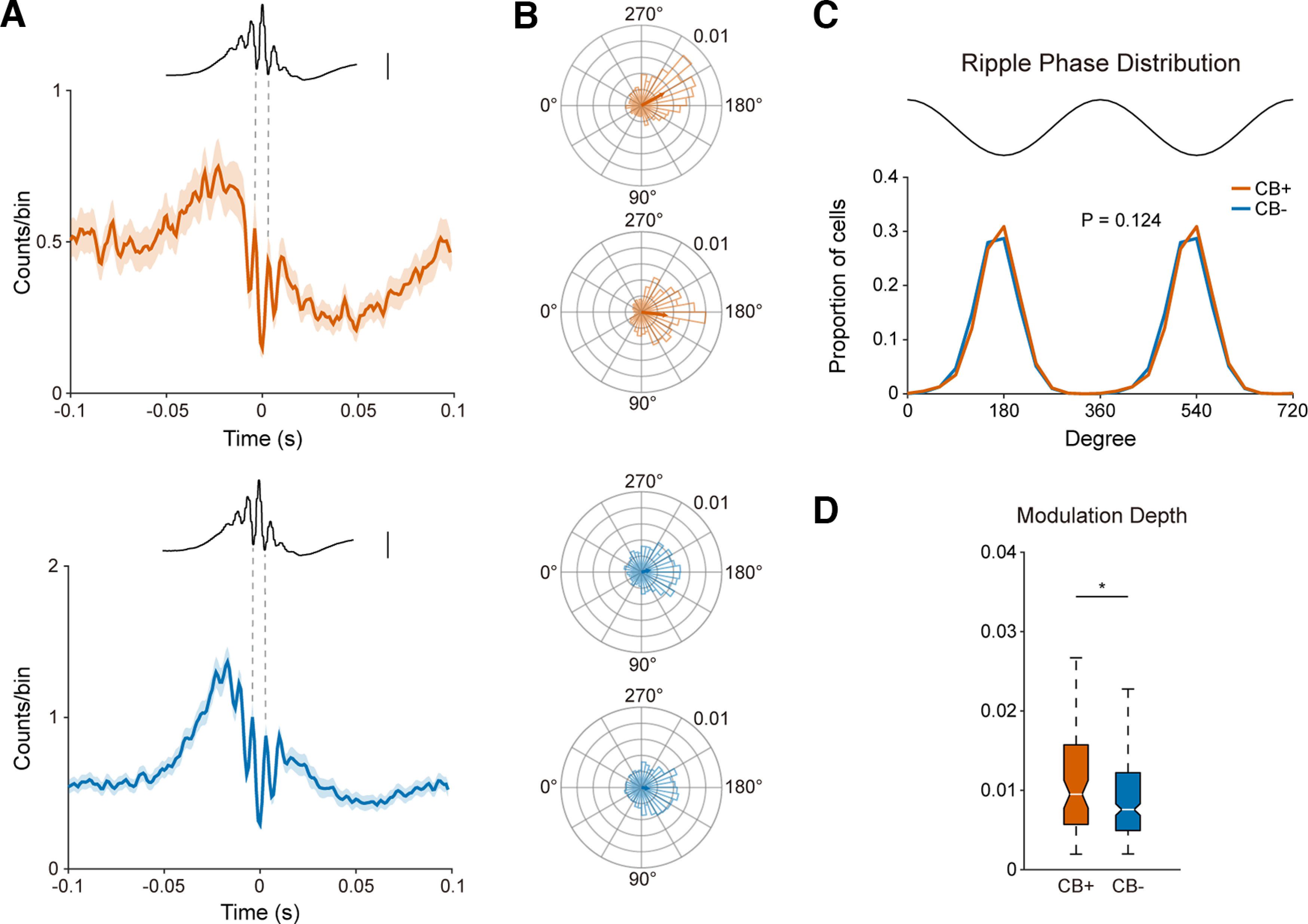
Both CB+ and CB− PNs are strongly Phase-locked to hippocampal ripple oscillations. ***A***, Phase-locked firing of both PN subtypes with hippocampal ripple oscillations, referenced at LFP ripple peak (orange, CB+ PNs, *n* = 116; blue, CB− PNs, *n* = 257). Averaged LFP ripple trace is illustrated at the top. Note that neuronal firings of both CB+ and CB− PNs are locked to LFP ripple troughs. Bin size: 1 ms. Scale bar: 0.25 mV. ***B***, Polar plots of neuronal firing phase distribution of example CB+ and CB− PNs. Bin size: 10°. ***C***, Population ripple phase distribution of both CB+ and CB− PNs (bin size: 30°, *p* = 0.124, Watson’s U^2^ test). Both PN subtypes are phase locked to ripple trough. ***D***, CB+ PNs are more strongly phase locked to ripple oscillations than CB− PNs (*p = 0.027, Mann–Whitney test).

Finally, we compared the modulation of ripple phase-locked firing between CB+ and CB− PNs during SWS. Neuronal firing activities of both PN subtypes were phase-locked to the trough of ripple oscillations with no significant phase-locking difference (Fig. 6*A–C*). Although CB− PNs are more actively engaged in ripples, CB+ PNs showed stronger phase-locked firing compared with CB− PNs (Fig. 6*D*).

## Discussion

In this study, we systematically investigated the *in vivo* firing patterns of two hippocampal PN subtypes based on Calbindin expression profile in free-moving mice. The subclassification of hippocampal pyramidal neurons by Calbindin expression profile may provide us with more precise classification and deeper understanding of the functional heterogeneity of hippocampal pyramidal neurons. We found that CB− PNs showed higher firing rates during running, while CB+ PNs displayed a higher tendency of burst firing regardless of behavior states. We also found that CB+ PNs can represent spatial information more efficiently: they are less likely to form place cells compared with their CB− counterparts, but with more spikes inside place fields. The firing of CB+ place cells also carry more information during spatial navigation. During REM sleep, a subset of CB+ PNs shifted their theta firing phase, while CB− PNs remained phase-locked to theta through. CB− PNs showed significantly higher participation in ripple oscillations during SWS.

### Using Calbindin as a molecular marker to differentiate hippocampal PNs

Calbindin has long been used as a molecular marker to identify neurons in the brain (Baimbridge and Miller, 1982; Sloviter, 1989). Recent studies have used the Calbindin expression profile to identify CB+ and CB− PNs with different afferents and neural circuits underlying different behaviors (Li et al., 2017; Pi et al., 2020). On the other hand, functional investigation of Calbindin, a Ca^2+^ binding protein, is scarce. One study has shown that Calbindin equips hippocampal neurons with mobile, high-affinity Ca^2+^-binding sites that slow and reduce global Ca^2+^ signals (Müller et al., 2005), indicating that Calbindin might reduce the activity of hippocampal pyramidal neurons. Another study recorded from dentate granule cells of Calbindin knock-out mice with patch clamp recording. They reported hyperexcitability of dentate gyrus granule cells in Calbindin knock-out mice (Kim et al., 2021). In our study, we used Calbindin as a molecular marker to distinguish superficial layer CB+ and deep layer CB− PNs. We found a significantly lower firing rate of CB+ PNs than that of CB− PNs during running (Fig. 2*B*) and hippocampal ripple oscillations (Fig. 5*E*). These results are consistent with the two aforementioned studies. The functional heterogeneity of CB+ and CB− PNs in our study could rise from their relative somatic location within the stratum pyramidale and hence their distinct afferents with other brain regions. Meanwhile, the physiological function of Calbindin may also be a contributing factor.

### The Calbindin expression dichotomy versus the superficial/deep dichotomy in neural coding heterogeneity

Neural coding heterogeneity of hippocampal deep and superficial layer pyramidal neurons have been reported recently (Mizuseki et al., 2011; Danielson et al., 2016; Fernández-Ruiz et al., 2021). Burst firings are different from single spikes in a way that they might play a different role in transmitting sensory information (Krahe and Gabbiani, 2004). We have shown that CB+ PNs showed a higher burst index regardless of behavior states, while Kenji Mizuseki found that more deep layer pyramidal neurons (presumably CB− PNs) showed higher burst index than superficial PNs (Mizuseki et al., 2011). We reasoned that on one hand, CB+ PNs receive more excitatory inputs from LEC and less inhibitory innervation from local parvalbumin positive basket cells (Lee et al., 2014; Li et al., 2017), which could lead to more burst spikes. On the other hand, CB− PNs showed a significantly higher firing rate during running, which as a denominator for calculating burst index, could result in significantly lower burst index during RUN.

Studies had shown similar spatial representation characteristics of deep and superficial layer pyramidal neurons including higher in/out place field firing rate ratio and spatial information per spike (Mizuseki et al., 2011; Fernández-Ruiz et al., 2021), which we confirmed in our comparison between CB+ and CB− PNs. The medial entorhinal cortex (MEC) is essential for spatial navigation of animals. A previous study has shown that strong MEC and weak LEC inputs favor deep PNs with more SLM spines in CA1c (close to CA2). In CA1a (near subiculum), strong LEC and weak MEC inputs favor superficial PNs with more SLM spines (Masurkar et al., 2017). Our previous study has shown that the MEC projects equally to CB+ and CB− PNs, while the lateral entorhinal cortex projects almost exclusively to CB+ PNs in dorsal CA1 (Li et al., 2017). In our experiments, we placed our recording electrodes in CA1b (mid-CA1) and found that CB+ PNs can represent spatial information more efficiently. The underlying mechanisms of such efficient spatial representation of CB+ PNs, e.g., the potential contribution of LEC and/or other brain areas that project differentially to CB+ and CB− PNs, is worth further investigation.

Interestingly, we found no significant difference in phase precession parameters between CB+ and CB− PNs, inconsistent with a recent study (Fernández-Ruiz et al., 2021). In our study, mice were trained to run freely back and forth in a U-shaped track, while others recorded from head-fixed mice running on a belt consisting of a cue-rich and cue-poor zones. The difference in self-motion and external visual cues between the two experiment setups may contribute to such controversy.

Theta oscillation dominates animal active running states, representing an online learning state (Buzsáki, 2002). During such exploration, neurons fire at specific phase of each theta cycle, representing distinct phase of information encoding process. Encoding occurs at the trough and rising slope of theta, when current sinks are strong in SLM, where entorhinal input terminates, and currents in layers receiving CA3 input are weak (Hasselmo, 2006). Indeed, we found that both CB+ and CB− PNs are phase-locked to trough of theta oscillations during running on the track, also consistent with previous report (Mizuseki et al., 2011). Theta oscillations were also prominent during REM sleeps. We found that a subset of CB+ PNs shifted their theta firing phase during REM sleep, CB− PNs remained the same theta firing phase as during running. Kenji Mizuseki had reported similar results with one subtype of PNs remained their theta firing phase while the other shifted their theta phase during REM sleep, except that they found the REM-shifting neurons were located in the deep layer of Stratum Pyramidale, presumably CB− PN subtypes. The somatic distribution of CB+ and CB− PNs may not follow the laminar distribution of deep and superficial layers (Baimbridge et al., 1991; Klausberger and Somogyi, 2008). In our study, optogenetic inhibition enabled us to identify CB+ and CB− PNs more accurately, thus allowing us to investigate the differences in their firing patterns more precisely.

### Distinct involvement of CB+ and CB− PNs during hippocampal ripple oscillations

Hippocampal ripple oscillations are high frequency transient network events recorded in the hippocampus during periods of immobility and slow wave sleep (Ylinen et al., 1995). Selective disruption of ripples during postlearning sleep results in impairment of behavior performance (Girardeau et al., 2009; Jadhav et al., 2012), indicating that hippocampal ripple oscillations are critical for memory consolidation. Pyramidal neurons in the hippocampus fire at specific phase of each ripple cycle (English et al., 2014; Hulse et al., 2016; Gan et al., 2017). The involvement of neuronal firing of different PN subtypes with ripple oscillations has not been fully addressed. In our study, we found that CB+ PNs are activated earlier during ripples than CB− PNs, while CB− PNs showed a significant higher participation in ripples than CB+ PNs. Previous report had pointed out hippocampal CA2 region as an initiation zone for sharp-wave ripples (SPW-R) in which the activity of CA2 neurons preceded SPW-R-related population activities in CA3 and CA1 (Oliva et al., 2016). Also, optogenetic activation of CA2 terminals results in significantly larger EPSC in deep layer CA1 pyramidal neurons than superficial layer neurons (Kohara et al., 2014). These observations might be the mechanism underlying the much stronger participation in ripple oscillations of CB− PNs than that of CB+ PNs. Another study had reported that hippocampal superficial layer pyramidal neurons are depolarized and increased their firing rate during ripple oscillations, while deep layer pyramidal neurons are hyperpolarized and decreased their firing rate during ripples (Valero et al., 2015). Distinct recording setups could be vital for such controversy of results, in which they recorded from head fixed rats under urethane anesthetization, while we recorded from free moving mice, which is closer to natural physiological states.

### Limitations of the study

There are a number of limitations of our study. First, the possibility that Arch expression in pyramidal neurons may affect the firing properties even in the absence of light stimulation. Based on the results of the first paper that introduced archaerhodopsin, the expression of Arch did not alter the basic cellular properties of the neuron, including membrane resistance, resting membrane potential, etc (Chow et al., 2010). We also examined potential change in neuronal firing properties because of prolonged photo-stimulation. We found no significant difference in population firing rate and action potential waveforms of CB+ and CB− PNs before and after photo-stimulation (data not shown).

Second, we used multichannel *in vivo* ephys recordings (tetrode) in our study, with the combination of optogenetics inhibition. There is certain limitation when categorizing pyramidal neurons. Some CB+ PNs might be misclassified as CB− PNs because of unsuccessful or insufficient expression of Arch, leading to a low light inhibition effect. On the other hand, CB− PNs could also be misclassified as CB+ PNs if they happened to not fire during the opto-stimulation period. Although the possibility is quite low, we still cannot quite exclude such possibility.

Third, all of our experiments were conducted in male mice. The findings of our study may not extend to females.

Overall, we investigated the basic firing pattern, representation of spatial information, and neuronal firing dynamics of pyramidal neuron subpopulation under hippocampal theta and ripple oscillations. We found prominent heterogeneity of neural activity between CB+ and CB− pyramidal neurons at single unit level, implying different roles in hippocampal neural code. Our results pointed out the necessity to pay attention to such functional heterogeneity between hippocampal pyramidal neurons in future investigations.

## Synthesis

Reviewing Editor: Christie Fowler, University of California, Irvine Decisions are customarily a result of the Reviewing Editor and the peer reviewers coming together and discussing their recommendations until a consensus is reached. When revisions are invited, a fact-based synthesis statement explaining their decision and outlining what is needed to prepare a revision will be listed below. The following reviewer(s) agreed to reveal their identity: Bruce McNaughton, Ryan LaLumiere. Note: If this manuscript was transferred from JNeurosci and a decision was made to accept the manuscript without peer review, a brief statement to this effect will instead be what is listed below.

This manuscript was reviewed by two experts in the field. Both felt that the investigations were of interest. It was noted that the findings reveal potentially important physiological differences in how the calbindin-expressing CA1 cells and non-expressing CA1 cells encode spatial information and then how this is replayed during sleep. However, significant methodological concerns were noted that preclude proper interpretation of the data. The main concern was that the photostimulation may lead to longer-lasting changes in cellular activity during the session. It is noted that the study provided continuous optogenetic stimulation 2-3 times per 30 min session, with each stimulation lasting 2-3 minutes. This raises concerns that recorded neuronal activity in the couple minutes after stimulation may have been contaminated by this prolonged Arch activation. Further concerns were noted regarding Arch altering the properties of these cells, which requires additional control studies to support the manuscript’s conclusions. Further concerns from each reviewer are indicated below:

Reviewer 1

The authors used genetically encoded Arch to identify a subclass of CA1 pyramidal cells that express the calcium binding protein Calbindin (CB). CB+ cells in the mouse CA1 are known to be highly concentrated in the superficial part of the pyramidal layer (adjacent to str. radiatum -e.g., https://doi.org/10.1016/j.jchemneu.2020.101745) and a number of studies have identified differences in morphology, connectivity and behavioral firing correlates between superficial and deep portions of str. pyramidale. As the authors note, “deep and superficial PNs showed significant differences in firing rate, phase-locked firing with hippocampal theta and ripple oscillations (Mizuseki et al., 2011; Stark et al., 2014;Danielson et al., 2016). A subset of deep PNs shifted their firing phase during REM sleep, while superficial PNs are consistently phase-locked with theta oscillation either during running or REM sleep (Mizuseki et al., 2011)”

CB+ and CB- cells were recorded during awake behavior on a linear track and during slow-wave and REM sleep. The authors show a number of interesting differences between the two classes. CB+ neurons had sparser firing with greater in-field/out-field firing ratios and correspondingly higher spatial information per spike. There was a left shift in mean firing rates during running but no difference during SWS or REM.

There are some discrepancies between the properties of CB+ neurons reported and similar studies which focused on the superficial-deep dichotomy without CB+ identification. The authors should address how their results agree/differ with the published results.

My main caveat with the paper is that there is no discussion of the possibility that expression of Arch in Pyramidal cells may affect their firing properties even in the absence of the light stimulation, or possible persistent effects (e.g., intracellular pH, Cl gradient etc.) of photostimulation. The authors need to present a convincing case that such effects are not present in order for me to accept their findings unequivocally.

Reviewer 2

The manuscript by Gu et al. examines the activity of CA1 pyramidal cells, broken down by whether they express calbindin, in spatial encoding both during active runs and during sleep. Overall, the evidence indicates that these two different types of pyramidal neurons display differences with regard to spatial encoding. In particular, those expressing calbindin appear to be “better” (i.e., more efficient, etc.) with regarding to spatial encoding than those not expressing calbindin. Overall, the methods appear sound and the writing is adequate, though it requires some additional editing (e.g., the methods should be in past tense, the Discussion should have clearer paragraph breaks). The analyses are complex and supportive of the conclusions. The findings should be of interest to a broader field interested in hippocampal functioning. My suggestions below largely focus on some additional points that would strengthen the manuscript.

1. The introduction discusses deep vs. superficial layers of CA1 and differences between them, but does not indicate which ones overlap with calbindin expression. This should be part of the intro. Additionally, it would be helpful to know the degree of separation vs. overlap between calbindin expression, or lack thereof, and superficial vs. deep layers. Related to this, it would be helpful if the authors made it clear (perhaps in the Discussion) whether they think the calbindin expression has any functional implications for the CA1 cells or whether it is simply a marker of whether a CA1 neuron is in deep or superficial layers. I mention this because it seems that the real argument that the authors are making is that the functional differences between the cells are simply a function of their CA1 location and, therefore, their inputs/outputs, etc.

2. Although the authors make a point that the superficial vs. deep layers receive differential input from the entorhinal cortex, they focus on the lateral entorhinal. In the case of spatial learning (like the current manuscript), it is strange that they do not mention how medial entorhinal inputs differ between the two cell types or layers, as the medial entorhinal cortex is the classic region with grid cells that is believed to be a key building block for place cells. Medial entorhinal inputs to the two layers should be discussed.

3. A schematic diagram of the CA1 region, with the CB+ and CB- cells clearly labeled, would be helpful. I would also suggest showing how different inputs are thought to relate to the different classes of cells.

4. Although the data clearly indicate differences between the CB+ and CB- cells in terms of their physiological activity, it should be acknowledged that the authors cannot fully confirm that every cell in each group truly belonged to that group. That is, due to the use of the optogenetic tagging procedure, some CB+ or CB- cells could have been misclassified. Additionally, do the authors consider the CB- cells to be one “group” of cells? Or could they themselves be a heterogeneous collection of CA1 pyramidal cells?

5. The intro and discussion refer to prior papers that found physiological differences between the deep-layer and superficial-layer CA1 cells. However, it is not clear how those prior papers separated them. Were these whole-cell recordings (i.e., where the authors could actually identify deep vs. superficial)? However, this seems unlikely for the Mizuseki work. This should be specified for the different references, as it seems that the method for identifying these different cells is important.

## Author Response

Reviewer 1

The authors used genetically encoded Arch to identify a subclass of CA1 pyramidal cells that express the calcium binding protein Calbindin (CB). CB+ cells in the mouse CA1 are known to be highly concentrated in the superficial part of the pyramidal layer (adjacent to str. radiatum -e.g., https://doi.org/10.1016/j.jchemneu.2020.101745) and a number of studies have identified differences in morphology, connectivity and behavioral firing correlates between superficial and deep portions of str. pyramidale. As the authors note, “deep and superficial PNs showed significant differences in firing rate, phase-locked firing with hippocampal theta and ripple oscillations (Mizuseki et al., 2011; Stark et al.,2014;Danielson et al., 2016). A subset of deep PNs shifted their firing phase during REM sleep, while superficial PNs are consistently phase-locked with theta oscillation either during running or REM sleep (Mizuseki et al., 2011)” CB+ and CB- cells were recorded during awake behavior on a linear track and during slow-wave and REM sleep. The authors show a number of interesting differences between the two classes. CB+ neurons had sparser firing with greater in-field/out-field firing ratios and correspondingly higher spatial information per spike. There was a left shift in mean firing rates during running but no difference during SWS or REM.

-> We thank the reviewer for his/her important comments. We have performed additional analyses to address the reviewer’s concern. Our point-by-point response can be found below.

There are some discrepancies between the properties of CB+ neurons reported and similar studies which focused on the superficial-deep dichotomy without CB+ identification. The authors should address how their results agree/differ with the

published results.

->Indeed, our results showed similarities and discrepancies with some published works.

We believe our study provides new insights into the functional heterogeneity of hippocampal pyramidal neurons along the radial axes. We have discussed the similarities and discrepancies between our study and some published work in the discussion. For example, we have pointed out some similarities, including firing rates and spatial information (lines 508-512), theta firing phase during running (lines 534 - 536), etc.

Moreover, we have discussed in depth how some of our findings differ from published results. For example, burst index (lines 496 - 505), phase precession (lines 523 - 528), and theta phase shift during REM sleep (lines 536 - 546), etc.

My main caveat with the paper is that there is no discussion of the possibility that expression of Arch in Pyramidal cells may affect their firing properties even in the absence of the light stimulation, or possible persistent effects (e.g., intracellular pH, Cl gradient etc.) of photostimulation. The authors need to present a convincing case that such effects are not present in order for me to accept their findings unequivocally.

->We thank the reviewer for pointing out such a fundamentally important issue. The expression of archaerhodopsin (Arch), a proton pump, in principle neurons in mice to inhibit neuronal firing has been widely accepted and utilized since its publication in 2010(Chow et al., 2010). As the paper illustrated, the expression of Arch did not alter the basic

cellular properties of the neuron, including membrane resistance, resting membrane 3 potential, etc.

However, as the reviewer suggested, prolonged photostimulation may cause possible persistent effects (changes in intracellular pH, Cl gradient, etc.). Based on our extracellular e-phys recording technique, we do not have access to intracellular pH and Chloride gradient. However, we were able to compare the population firing rate of CB+ PNs before and after photostimulation (note that the data representing the “after photostimulation” was collected the next day at the beginning of the recording session).

As illustrated in the figure below (Figure1), we didn’t find a significant difference in population firing rate of CB+ PNs upon photostimulation under different behavior states.

Figure 1. The population firing rate of CB+ PNs is consistent before and after photostimulation. RUN: n = 56 before, n = 28 after photostimulation, p = 0.68, Man Whitney test; SWS: n = 77 before, n = 113 after photostimulation, p = 0.082, Man Whitney test; REM: n = 10 before, n = 60 after photostimulation, p = 0.079, Man Whitney test.

Furthermore, we reasoned that if something would happen to CB+ PNs upon photostimulation, we might see some change in the spike waveforms of CB+ PNs. So we compared the waveforms of CB+ PNs before and after photostimulation (Figure 2).

Shown here are average waveforms of 5 pyramidal neurons recorded from 4 tetrodes under running and SWS. Note that one neuron (SIG029b) was illustrated in both states, also demonstrating the recording stability of our method. We found that the waveforms from neuronal firing are almost identical before and after photostimulation.

4

Figure 2. Comparison of Neuronal firing waveforms of CB+ PNs before (red) and after(yellow) photostimulation under running (top row) and slow wave sleep state (bottom row). Waveform correlation values are illustrated at the top right corner of each neuron.

Lastly, we compared the firing rate of CA1 pyramidal neurons recorded from wild-type mice with the data reported in our manuscript (Figure 3, neuronal firing rate of CB+ PNs were also presented here for comparison). The ephys recording environment, recording technique, and protocol, were exactly the same between WT and transgenic mice. There was no significant difference in population firing rate between WT and transgenic mice under the three behavior states (RUN, red; SWS, blue; REM, cyan color in Figure 3).

CB+ PNs showed a significant lower firing rate compared to WT group during running (n = 127 for WT, and n = 62 for CB+ PNs, p = 0.025, Dunn’s multiple comparisons test).

5

Figure 3. Neuronal population firing rate comparison between neurons recorded from wild type mice (WT), transgenic mice (Cre), and CB+ PNs during three different behavior states. The numbers in parentheses indicate number of neurons in each group.

We hope that we have made a convincing case that potential persistent effects of the expression of Arch in pyramidal cells and also photostimulation are not present in our study. We conclude that neither the expression of Arch nor stimulation of these cells introduced significant persistent effects.

6

Reviewer 2

The manuscript by Gu et al. examines the activity of CA1 pyramidal cells, broken down by whether they express calbindin, in spatial encoding both during active runs and during sleep. Overall, the evidence indicates that these two different types of pyramidal neurons display differences with regard to spatial encoding. In particular, those expressing calbindin appear to be “better” (i.e., more efficient, etc.) with regarding to spatial encoding than those not expressing calbindin. Overall, the methods appear sound and the writing is adequate, though it requires some additional editing (e.g., the methods should be in past tense, the Discussion should have clearer paragraph breaks). The analyses are complex and supportive of the conclusions. The findings should be of interest to a broader field interested in hippocampal functioning. My suggestions below largely focus on some additional points that would strengthen the manuscript.

->We would like to thank the reviewer for their recommendation and suggestions for improvement. We have worked to address each of the concerns and made changes accordingly in the revised manuscript.

1. The introduction discusses deep vs. superficial layers of CA1 and differences between them, but does not indicate which ones overlap with calbindin expression. This should be part of the intro. Additionally, it would be helpful to know the degree of separation vs. overlap between calbindin expression, or lack thereof, and superficial vs. deep layers.

Related to this, it would be helpful if the authors made it clear (perhaps in the Discussion)whether they think the calbindin expression has any functional implications for the CA1 cells or whether it is simply a marker of whether a CA1 neuron is in deep or superficial layers. I mention this because it seems that the real argument that the authors are making is that the functional differences between the cells are simply a function of their CA1 location and, therefore, their inputs/outputs, etc.

->First of all, we thank the reviewer for his/her in-depth review of the manuscript and for pointing out this issue. We are sorry for not making it clear in the submitted manuscript.

Most neurons in the superficial layer of the dorsal CA1 area express Calbindin, while deep layer PNs did not. To what extent Calbindin expression overlaps with superficial and deep layer PNs is not clear and is beyond the scope of this study. We have added these descriptions appropriately in the introduction (lines 72-74).

Secondly, we took a deep dive into the literature to find out whether calbindin expression has any functional implications in CA1 or whether it is simply a marker of whether a CA1 neuron is in deep or superficial layers. Calbindin has long been used as a molecular marker to identify neurons in the brain (Baimbridge and Miller, 1982; Sloviter, 1989).

Recent studies have utilized the Calbindin expression profile to identify CB+ and CB- PNs with different afferents and neural circuits underlying different behaviors (Li et al.,2017; Pi et al., 2020). On the other hand, functional investigation of Calbindin, a Ca2+ binding protein, is scarce. One study has shown that “Calbindin equips hippocampal neurons with mobile, high-affinity Ca2+-binding sites that slow and reduce global Ca2+ signals.” (Müller et al., 2005), indicating that Calbindin might reduce the activity of hippocampal pyramidal neurons. Another study recorded from dentate granule cells of Calbindin knockout mice with patch clamp recording. They reported hyperexcitability of dentate gyrus granule cells in Calbindin knockout mice (Kim et al., 2021).

7

In our study, we used Calbindin as a molecular marker to distinguish superficial layer CB+ and deep layer CB- PNs. We found a significantly lower firing rate of CB+ PNs than that of CB- PNs during running (figure 2B) and hippocampal ripple oscillations (figure 5E). These results are consistent with the two aforementioned studies. Overall, the functional heterogeneity of CB+ and CB- PNs in our study could rise from their relative somatic location within the stratum pyramidale and hence their distinct afferents with other brain regions. Meanwhile, the physiological function of Calbindin may also be a contributing factor.

We think this would indeed strengthen the manuscript. We have added these paragraphs in the discussion (lines 480 - 498).

2. Although the authors make a point that the superficial vs. deep layers receive differential input from the entorhinal cortex, they focus on the lateral entorhinal. In the case of spatial learning (like the current manuscript), it is strange that they do not mention how medial entorhinal inputs differ between the two cell types or layers, as the medial entorhinal cortex is the classic region with grid cells that is believed to be a key building block for place cells. Medial entorhinal inputs to the two layers should be discussed.

->The medial entorhinal cortex (MEC) is essential for spatial navigation of animals. A previous study has shown that strong MEC and weak LEC inputs favor deep PNs with more SLM spines in CA1c (close to CA2). In CA1a (near subiculum), strong LEC and weak MEC inputs favor superficial PNs with more SLM spines(Masurkar et al., 2017). Our previous study has shown that the MEC projects equally to CB+ and CB- PNs, while the lateral entorhinal cortex projects almost exclusively to CB+ PNs in dorsal CA1(Li et al., 2017). In our experiments in the proposed manuscript, we placed our recording electrodes in CA1b (mid-CA1) and found that CB+ PNs can represent spatial information more efficiently. The underlying mechanisms of such efficient spatial representation of CB+ PNs, e.g., the potential contribution of LEC and/or other brain areas that project differentially to CB+ and CB- PNs, is worth further investigation.

We have also added this paragraph to the discussion (lines 515 - 525).

3. A schematic diagram of the CA1 region, with the CB+ and CB- cells clearly labeled, would be helpful. I would also suggest showing how different inputs are thought to relate to the different classes of cells.

-> We thank the reviewer for the suggestion. Following this direction, we have drawn a diagram focusing primarily on the connection between the LEC, MEC, and CA1 PNs based on our previous study (Li et al., 2017). The lateral entorhinal cortex projects almost exclusively to the superficial layer CB+ PNs, while the medial entorhinal cortex projects to both CB+ and CB- PNs equally. We have also mentioned some studies regarding different inputs to the different classes of cells including CA2 and local Parvalbumin inputs in the introduction (lines 76 - 84).

We agree with the reviewer that a schematic diagram of the CA1 region, with the CB+ and CB- cells clearly labeled, would be helpful. However, after intense discussion between the authors, we decided not to include such a diagram in our manuscript because these results have been published previously and are not the main focus of our proposed manuscript.

8

4. Although the data clearly indicate differences between the CB+ and CB- cells in terms of their physiological activity, it should be acknowledged that the authors cannot fully confirm that every cell in each group truly belonged to that group. That is, due to the use of the optogenetic tagging procedure, some CB+ or CB- cells could have been misclassified. Additionally, do the authors consider the CB- cells to be one “group” of cells? Or could they themselves be a heterogeneous collection of CA1 pyramidal cells?

->Indeed, as the reviewer pointed out, there was a certain flaw in our method with the optotagging technique. Some CB+ PNs might be misclassified as CB- PNs due to unsuccessful or insufficient expression of Arch, leading to a low light inhibition effect.

On the other hand, CB- PNs could also be misclassified as CB+ PNs if they happened to not fire during the opto-stimulation period. Although the possibility of this situation is quite low, we still can’t quite exclude such possibility. In our proposed manuscript, we calculated a light inhibition index for each neuron to represent the effect of opto-inhibition. Neurons with an inhibition index greater than 80% was labelled as CB+ PNs, and the rest CB- PNs. Despite the technical limitation of our method leading to some misclassification, we believe that most of the neurons in our dataset were correctly labelled as CB+ or CB- PNs.

Additionally, we do consider CB- PNs in our dataset to be one “group” of cells since they didn’t show any heterogeneous firing pattern in any of our analyses. CB+ PNs, however, showed a bimodal distribution of theta firing phase during REM sleep compared to running, which could indicate that CB+ PNs might be a heterogeneous collection of neurons.

5. The intro and discussion refer to prior papers that found physiological differences between the deep-layer and superficial-layer CA1 cells. However, it is not clear how those prior papers separated them. Were these whole-cell recordings (i.e., where the authors could actually identify deep vs. superficial)? However, this seems unlikely for the Mizuseki work. This should be specified for the different references, as it seems that the method for identifying these different cells is important.

->In the Mizuseki work, extracellular ephys recordings were performed in free-moving rats with 4- or 8- shank silicon probes, with 8 recording sites on each shank. Superficial and deep layer pyramidal neurons are identified based on extracellular ephys characteristics. To be specific, among the 8 recording sites, the one with the largest amplitude of ripples was considered the middle of the pyramidal layer and served as the reference depth for calculating the distance of the recorded cell bodies from the middle of the pyramidal layer. Based on this relative distance, each pyramidal neuron was assigned to the superficial or deep layer group, with the superficial layer close to stratum radiatum,

9

while deep layer close to stratum oriens (Mizuseki et al., 2011).

We have specified the method for identifying different cells for different referencesaccordingly in the revised manuscript (lines 86 - 89).

Reference:

1. Baimbridge KG, Miller JJ (1982) Immunohistochemical localization of calcium-binding protein in the cerebellum, hippocampal formation and olfactory bulb of the rat. Brain research 245:223-229.

2. Chow BY, Han X, Dobry AS, Qian X, Chuong AS, Li M, Henninger MA, Belfort GM, Lin Y, Monahan PE, Boyden ES (2010) High-performance genetically targetable optical neural silencing by light-driven proton pumps. Nature 463:98-102.

3. Kim KR, Jeong HJ, Kim Y, Lee SY, Kim Y, Kim HJ, Lee SH, Cho H, Kang JS, Ho WK (2021) Calbindin regulates Kv4.1 trafficking and excitability in dentate granule cells via CaMKII-dependent phosphorylation. Exp Mol Med 53:1134-1147.

4. Li Y, Xu J, Liu Y, Zhu J, Liu N, Zeng W, Huang N, Rasch MJ, Jiang H, Gu X, Li X, Luo M, Li C, Teng J, Chen J, Zeng S, Lin L, Zhang X (2017) A distinct entorhinal cortex to hippocampal CA1 direct circuit for olfactory associative learning. Nature neuroscience 20:559-570.

5. Masurkar AV, Srinivas KV, Brann DH, Warren R, Lowes DC, Siegelbaum SA (2017) Medial and Lateral Entorhinal Cortex Differentially Excite Deep versus Superficial CA1 Pyramidal Neurons. Cell reports 18:148-160.

6. Mizuseki K, Diba K, Pastalkova E, Buzsaki G (2011) Hippocampal CA1 pyramidal cells form functionally distinct sublayers. Nature neuroscience 14:1174-1181.

7. Müller A, Kukley M, Stausberg P, Beck H, Müller W, Dietrich D (2005) Endogenous Ca2+ buffer concentration and Ca2+ microdomains in hippocampal neurons. The Journal of neuroscience : the official journal of the Society for Neuroscience 25:558-565.

8. Pi G et al. (2020) Posterior basolateral amygdala to ventral hippocampal CA1 drives approach behaviour to exert an anxiolytic effect. Nature communications 11:183.

9. Sloviter RS (1989) Calcium-binding protein (calbindin-D28k) and parvalbumin immunocytochemistry: localization in the rat hippocampus with specific reference to the selective vulnerability of hippocampal neurons to seizure activity. J Comp Neurol 280:183-196.
